# *N*-[3-(2,6-Dimethylanilino)-1-methylbut-2-enylidene]-2,6-dimethylanilinium chloride^1^

**DOI:** 10.1107/S1600536809020571

**Published:** 2009-06-24

**Authors:** Víctor M. Jiménez-Pérez, Sylvain Bernès, Blanca M. Munõz, Boris I. Kharisov, Andrea V. Vela

**Affiliations:** a Facultad de Ciencias Químicas, Universidad Autónoma de Nuevo León, Av. Pedro de Alba s/n, 66451 San Nicolás de los Garza, N. L., Mexico; b CINVESTAV-Monterrey, Vía del conocimiento 201, PIIT. Autopista al Aeropuerto Km. 9.5, Apodaca, N. L. Mexico

## Abstract

The title salt, C_21_H_27_N_2_^+^·Cl^−^ resulted from the condensation between 2,6-dimethyl­aniline and acetyl­acetone in acidified ethanol. The bulky cation is stabilized in a *β*-imino­enamine tautomeric form, and presents a W-shaped conformation. The benzene rings are arranged almost parallel, with a dihedral angle of 6.58 (4)° between the mean planes. Both N—H groups in the cation form strong hydrogen bonds with two symmetry-related chloride anions. The resulting supra­molecular structure is a one dimensional polymer running along [001], alternating cations and anions. The π–π inter­action observed in the mol­ecule, characterized by a centroid–centroid separation of 4.298 (4) Å, is thus extended to the chains, with separations of 5.222 (4) Å between benzene rings of neighbouring cations in the crystal.

## Related literature

For the synthesis, properties, and uses of *β*-diketimines and *β*-diketiminates, see: Dorman (1966 ▶); Park (2007 ▶); Bourget-Merle *et al.* (2002 ▶); Nagendran & Roesky (2008 ▶); Holland & Tolman (2000 ▶); Stender *et al.* (2001 ▶); Carey *et al.*, 2003 ▶. For W-shaped cations related to the title compound, see: Brownstein *et al.* (1983 ▶); Kuhn *et al.* (2000 ▶); Lesikar & Richards (2006 ▶).
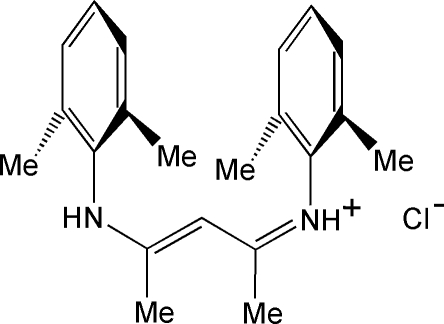


## Experimental

### 

#### Crystal data

C_21_H_27_N_2_^+^·Cl^−^
*M* *_r_* = 342.90
Tetragonal, 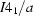

*a* = 28.639 (5) Å
*c* = 10.150 (3) Å
*V* = 8325 (3) Å^3^
*Z* = 16
Mo *K*α radiation
μ = 0.19 mm^−1^
*T* = 298 K
0.50 × 0.36 × 0.22 mm

#### Data collection

Bruker P4 diffractometer
Absorption correction: ψ scan *XSCANS* (Siemens, 1996 ▶) *T*_min_ = 0.854, *T*_max_ = 0.959
7193 measured reflections
3671 independent reflections
1541 reflections with *I* > 2σ(*I*)
*R*_int_ = 0.049
3 standard reflections every 97 reflections intensity decay: 1.5%

#### Refinement

*R*[*F* ^2^ > 2σ(*F* ^2^)] = 0.062
*wR*(*F* ^2^) = 0.171
*S* = 1.12
3671 reflections
229 parameters
H atoms treated by a mixture of independent and constrained refinement
Δρ_max_ = 0.43 e Å^−3^
Δρ_min_ = −0.32 e Å^−3^

### 

Data collection: *XSCANS* (Siemens, 1996 ▶); cell refinement: *XSCANS*; data reduction: *XSCANS*; program(s) used to solve structure: *SHELXS97* (Sheldrick, 2008 ▶); program(s) used to refine structure: *SHELXL97* (Sheldrick, 2008 ▶); molecular graphics: *Mercury* (Macrae *et al.*, 2006 ▶); software used to prepare material for publication: *SHELXL97*.

## Supplementary Material

Crystal structure: contains datablocks I, global. DOI: 10.1107/S1600536809020571/fl2243sup1.cif

Structure factors: contains datablocks I. DOI: 10.1107/S1600536809020571/fl2243Isup2.hkl

Additional supplementary materials:  crystallographic information; 3D view; checkCIF report

## Figures and Tables

**Table 1:** Hydrogen-bond geometry (Å, °)

*D*—H⋯*A*	*D*—H	H⋯*A*	*D*⋯*A*	*D*—H⋯*A*
N11—H11⋯Cl1	1.01 (3)	2.12 (3)	3.115 (3)	170 (3)
N7—H7⋯Cl1^i^	1.00 (3)	2.12 (3)	3.110 (4)	170 (3)

Symmetry code: (i) 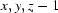.

**Fig. 1.**
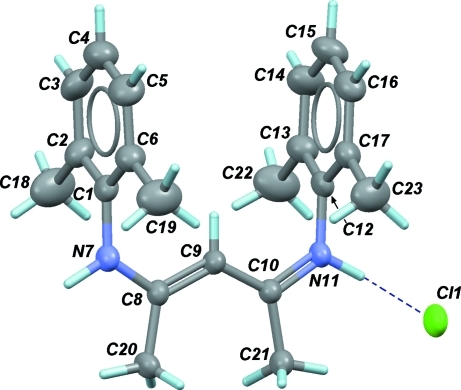
The title compound (asymmetric unit) with displacement ellipsoids at the 25% probability level. The dashed bond corresponds to the N—H···Cl hydrogen bond.

**Fig. 2.**
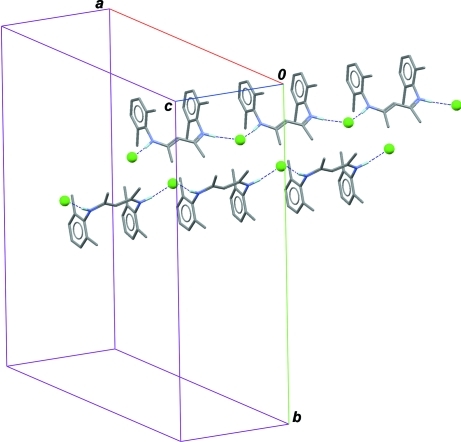
A part of the crystal structure of the title compound, with hydrogen bonds displayed as dashed lines. Two neighbouring chains are shown, with Cl- ions represented as green spheres, and omitting all C-bonded H atoms.

## supplementary crystallographic information

### Comment

Many synthetic routes are currently available to synthesize symmetric and unsymmetric *β*-diketimines, including the co-condensation reaction of a ketone and a primary amine (Dorman, 1966; Park, 2007). These ligands proved to be very versatile and diverse, considering the possible variation of both coordination modes and of groups bonded to the N atoms and to the *α*- and *β*- C atoms. Indeed, *β*-diketiminates are the most used ligands in coordination chemistry for the stabilization of low coordinate and low oxidation states of main group or transition elements (Bourget-Merle *et al.*, 2002; Nagendran & Roesky, 2008). *β*-Diketiminates complexes have been used as catalysts and even as structural models of protein active sites (Holland & Tolman, 2000).

Neutral *β*-diketimines invariably show a U-shaped conformation, favoured over other possible conformers by the formation of an intramolecular hydrogen bond involving the amine and imine N atoms (Dorman, 1966; Stender *et al.*, 2001; Carey *et al.*, 2003). However, if the *β*-diketimine is protonated, the backbone molecule adopts a new W-conformation, since the protonated N atom is able to form a strong intra-ionic N—H···Cl bond. Such behaviour has been observed in three salts closely related to the title compound where phenyl (Brownstein *et al.*, 1983), methyl (Kuhn *et al.*, 2000), and mesityl (Lesikar & Richards, 2006) have replaced the  2,6-dimethylphenyl. The supramolecular one-dimensional structure generated by this hydrogen bond is expected to be a strong stabilizing factor, as anions and cations alternate in a chain structure, which has a theoretical Madelung constant of 1.38.

In the title salt, the cation adopts the W-shaped conformation, and thus presents a non-crystallographic twofold axis passing through the central atom C9 (Fig. 1). Both N atoms are protonated, indicating that the *β*-iminoenamine tautomeric form is stabilized in the solid state. A parallel arrangement is observed for benzene rings, which are separated by 4.298 (4) Å. A similar arrangement was found with the mesityl-including cation, although the benzene separations are larger, probably because of the hindering character of mesityl (centroid-to-centroid distances: 4.348, 4.823, or 4.881 Å, depending of the nature of the counterion; Lesikar & Richards, 2006). Unexpectedly, the phenyl-containing salt has little intramolecular π–π interaction, with non-parallel phenyl rings separated by 5.480 Å (Brownstein *et al.*, 1983). In the title cation, benzene rings are close to be parallel, the dihedral angle between mean planes being 6.58 (4)°.

Regarding the crystal structure, both amine and imine NH functionalities, N7 and N11, are involved in strong N—H···Cl hydrogen bonds with symmetry-related Cl^-^ ions. The network of hydrogen bonds forms a one-dimensional supramolecular structure along the short cell axis *c* (Fig. 2). Cations and anions alternate in a chain, with all benzene rings oriented in the same direction. This arrangement allows to extend the π–π interactions to the whole polymeric structure, with a separation of 5.222 (4) Å between benzene rings of neighbouring cations. No significant contacts are observed between chains in the crystal.

### Experimental

The title salt was prepared by mixing acetylacetone (25.03 g, 0.25 mol) and 2,6-dimethylaniline (60.5 g, 0.5 mol) in 12 N hydrochloric acid (20.8 ml, 0.25 mol HCl). The mixture was heated to 393 K for 4 h., allowing the water to distil. The reaction was then further heated to 413 K overnight. The resulting solid was recrystallized from hot ethanol, yielding 87.6 g of the title salt (94%). NMR data are in agreement with the X-ray structure (see archived CIF).

### Refinement

All C-bonded H atoms were placed in calculated positions and refined as riding to their carrier atoms, with bond lengths fixed to 0.93 (aromatic CH) or 0.96 Å (methyl CH_3_). N-bonded H atoms (H7 and H11) were found in a difference map and refined freely. Isotropic displacement parameters for H atoms were calculated as *U*_iso_(H) = 1.5*U*_eq_(carrier atom) for methyl groups and *U*_iso_(H) = 1.2*U*_eq_(carrier atom) otherwise.

### Crystal data

**Table:**

C_21_H_27_N_2_^+^·Cl^−^	*D*_x_ = 1.094 Mg m^−^^3^
*M**_r_* = 342.90	Mo *K*α radiation, λ = 0.71073 Å
Tetragonal, *I*4_1_/*a*	Cell parameters from 77 reflections
Hall symbol: -I 4ad	θ = 4.6–12.3°
*a* = 28.639 (5) Å	µ = 0.19 mm^−^^1^
*c* = 10.150 (3) Å	*T* = 298 K
*V* = 8325 (3) Å^3^	Prism, colorless
*Z* = 16	0.50 × 0.36 × 0.22 mm
*F*(000) = 2944	

### Data collection

**Table:**

Bruker P4 diffractometer	1541 reflections with *I* > 2σ(*I*)
Radiation source: fine-focus sealed tube	*R*_int_ = 0.049
graphite	θ_max_ = 25.1°, θ_min_ = 2.0°
ω scans	*h* = −34→13
Absorption correction: ψ scan *XSCANS* (Siemens, 1996)	*k* = −34→1
*T*_min_ = 0.854, *T*_max_ = 0.959	*l* = −12→12
7193 measured reflections	3 standard reflections every 97 reflections
3671 independent reflections	intensity decay: 1.5%

### Refinement

**Table:**

Refinement on *F*^2^	Primary atom site location: structure-invariant direct methods
Least-squares matrix: full	Secondary atom site location: difference Fourier map
*R*[*F*^2^ > 2σ(*F*^2^)] = 0.062	Hydrogen site location: inferred from neighbouring sites
*wR*(*F*^2^) = 0.171	H atoms treated by a mixture of independent and constrained refinement
*S* = 1.12	*w* = 1/[σ^2^(*F*_o_^2^) + (0.06*P*)^2^] where *P* = (*F*_o_^2^ + 2*F*_c_^2^)/3
3671 reflections	(Δ/σ)_max_ < 0.001
229 parameters	Δρ_max_ = 0.43 e Å^−^^3^
0 restraints	Δρ_min_ = −0.31 e Å^−^^3^
0 constraints	

### Fractional atomic coordinates and isotropic or equivalent isotropic displacement parameters (Å^2^)

**Table:**

	*x*	*y*	*z*	*U*_iso_*/*U*_eq_	
Cl1	0.89962 (3)	0.22980 (3)	0.87464 (9)	0.0839 (4)	
C1	0.84317 (12)	0.14527 (11)	0.1392 (5)	0.0623 (9)	
C2	0.79611 (13)	0.15570 (13)	0.1335 (6)	0.0824 (11)	
C3	0.76486 (14)	0.11910 (17)	0.1272 (7)	0.1044 (14)	
H3A	0.7330	0.1251	0.1223	0.125*	
C4	0.78047 (16)	0.07434 (16)	0.1282 (8)	0.1031 (13)	
H4A	0.7591	0.0499	0.1251	0.124*	
C5	0.82605 (17)	0.06477 (14)	0.1334 (7)	0.1001 (13)	
H5A	0.8360	0.0339	0.1322	0.120*	
C6	0.85857 (13)	0.10015 (13)	0.1404 (6)	0.0800 (12)	
N7	0.87572 (10)	0.18264 (10)	0.1423 (4)	0.0623 (8)	
H7	0.8870 (13)	0.1961 (13)	0.057 (3)	0.075*	
C8	0.89275 (12)	0.20174 (12)	0.2519 (3)	0.0528 (9)	
C9	0.87894 (9)	0.18619 (10)	0.3745 (3)	0.0565 (8)	
H9A	0.8571	0.1622	0.3753	0.068*	
C10	0.89372 (12)	0.20212 (12)	0.4965 (3)	0.0527 (9)	
N11	0.87729 (10)	0.18288 (10)	0.6051 (3)	0.0592 (8)	
H11	0.8875 (13)	0.1952 (12)	0.693 (3)	0.071*	
C12	0.84451 (12)	0.14556 (11)	0.6106 (5)	0.0589 (9)	
C13	0.79765 (13)	0.15614 (12)	0.6194 (7)	0.0801 (10)	
C14	0.76658 (13)	0.11931 (15)	0.6254 (7)	0.1053 (13)	
H14A	0.7347	0.1253	0.6284	0.126*	
C15	0.78209 (15)	0.07445 (14)	0.6271 (7)	0.0982 (12)	
H15A	0.7607	0.0501	0.6335	0.118*	
C16	0.82809 (16)	0.06482 (12)	0.6197 (6)	0.0886 (11)	
H16A	0.8380	0.0339	0.6192	0.106*	
C17	0.86083 (12)	0.10022 (12)	0.6127 (6)	0.0725 (10)	
C18	0.78029 (14)	0.20557 (14)	0.1303 (8)	0.144 (2)	
H18A	0.7929	0.2219	0.2049	0.217*	
H18B	0.7468	0.2067	0.1336	0.217*	
H18C	0.7910	0.2201	0.0506	0.217*	
C19	0.90988 (14)	0.08956 (14)	0.1468 (7)	0.130 (2)	
H19A	0.9144	0.0564	0.1496	0.195*	
H19B	0.9230	0.1035	0.2245	0.195*	
H19C	0.9251	0.1021	0.0702	0.195*	
C20	0.92742 (11)	0.23930 (11)	0.2268 (3)	0.0681 (10)	
H20A	0.9303	0.2443	0.1336	0.102*	
H20B	0.9572	0.2303	0.2622	0.102*	
H20C	0.9172	0.2676	0.2683	0.102*	
C21	0.92784 (11)	0.23993 (11)	0.5203 (3)	0.0685 (10)	
H21A	0.9306	0.2454	0.6134	0.103*	
H21B	0.9174	0.2680	0.4777	0.103*	
H21C	0.9577	0.2310	0.4854	0.103*	
C22	0.78164 (14)	0.20619 (14)	0.6190 (8)	0.1295 (17)	
H22A	0.7974	0.2230	0.6876	0.194*	
H22B	0.7486	0.2073	0.6338	0.194*	
H22C	0.7887	0.2201	0.5354	0.194*	
C23	0.91199 (13)	0.08983 (13)	0.6062 (7)	0.1174 (17)	
H23A	0.9168	0.0567	0.6127	0.176*	
H23B	0.9277	0.1052	0.6778	0.176*	
H23C	0.9244	0.1009	0.5241	0.176*	

### Atomic displacement parameters (Å^2^)

**Table:**

	*U*^11^	*U*^22^	*U*^33^	*U*^12^	*U*^13^	*U*^23^
Cl1	0.1080 (7)	0.1037 (7)	0.0400 (4)	−0.0103 (5)	−0.0002 (6)	0.0004 (6)
C1	0.077 (2)	0.063 (2)	0.047 (2)	−0.0056 (19)	−0.003 (2)	−0.007 (2)
C2	0.084 (3)	0.076 (2)	0.087 (3)	−0.002 (2)	−0.023 (4)	−0.011 (4)
C3	0.081 (3)	0.119 (3)	0.113 (3)	−0.013 (3)	−0.034 (5)	0.002 (7)
C4	0.106 (3)	0.098 (3)	0.105 (3)	−0.028 (3)	0.000 (6)	0.001 (6)
C5	0.121 (3)	0.075 (3)	0.105 (3)	−0.007 (3)	0.000 (5)	−0.023 (4)
C6	0.089 (3)	0.068 (2)	0.083 (3)	−0.009 (2)	−0.006 (3)	−0.003 (3)
N7	0.0791 (19)	0.0624 (17)	0.045 (2)	−0.0064 (15)	−0.0018 (18)	−0.0033 (18)
C8	0.062 (2)	0.054 (2)	0.0422 (19)	0.0017 (18)	−0.0027 (17)	−0.0015 (17)
C9	0.0653 (18)	0.0616 (18)	0.0426 (15)	−0.0103 (14)	−0.0007 (19)	−0.0040 (19)
C10	0.060 (2)	0.052 (2)	0.045 (2)	−0.0028 (17)	−0.0009 (17)	−0.0006 (17)
N11	0.0787 (18)	0.0613 (17)	0.038 (2)	−0.0112 (14)	−0.0008 (17)	0.0027 (16)
C12	0.073 (2)	0.064 (2)	0.040 (2)	−0.0079 (19)	0.006 (2)	0.004 (2)
C13	0.085 (3)	0.072 (2)	0.082 (3)	−0.009 (2)	0.011 (4)	−0.001 (4)
C14	0.078 (3)	0.106 (3)	0.132 (4)	−0.013 (3)	0.007 (5)	0.015 (7)
C15	0.105 (3)	0.082 (3)	0.107 (3)	−0.021 (2)	0.025 (5)	0.011 (5)
C16	0.118 (3)	0.068 (2)	0.079 (3)	−0.007 (2)	0.009 (5)	−0.005 (4)
C17	0.084 (2)	0.066 (2)	0.067 (3)	−0.007 (2)	−0.006 (3)	0.011 (3)
C18	0.108 (3)	0.104 (3)	0.221 (6)	0.025 (3)	−0.046 (7)	−0.006 (7)
C19	0.103 (3)	0.092 (3)	0.194 (6)	0.016 (2)	−0.004 (5)	−0.012 (5)
C20	0.087 (2)	0.070 (2)	0.0476 (18)	−0.017 (2)	0.010 (2)	0.0013 (17)
C21	0.083 (2)	0.072 (2)	0.0499 (19)	−0.013 (2)	−0.006 (2)	0.0015 (18)
C22	0.103 (3)	0.095 (3)	0.190 (5)	0.017 (2)	0.028 (7)	−0.001 (6)
C23	0.096 (3)	0.092 (3)	0.164 (5)	0.013 (2)	−0.022 (4)	0.006 (4)

### Geometric parameters (Å, °)

**Table:**

C1—C6	1.366 (4)	C14—C15	1.360 (5)
C1—C2	1.382 (5)	C14—H14A	0.9300
C1—N7	1.419 (4)	C15—C16	1.348 (5)
C2—C3	1.380 (5)	C15—H15A	0.9300
C2—C18	1.499 (5)	C16—C17	1.383 (4)
C3—C4	1.358 (5)	C16—H16A	0.9300
C3—H3A	0.9300	C17—C23	1.497 (4)
C4—C5	1.335 (5)	C18—H18A	0.9600
C4—H4A	0.9300	C18—H18B	0.9600
C5—C6	1.378 (5)	C18—H18C	0.9600
C5—H5A	0.9300	C19—H19A	0.9600
C6—C19	1.502 (5)	C19—H19B	0.9600
N7—C8	1.332 (4)	C19—H19C	0.9600
N7—H7	1.00 (3)	C20—H20A	0.9600
C8—C9	1.379 (4)	C20—H20B	0.9600
C8—C20	1.486 (4)	C20—H20C	0.9600
C9—C10	1.386 (4)	C21—H21A	0.9600
C9—H9A	0.9300	C21—H21B	0.9600
C10—N11	1.319 (4)	C21—H21C	0.9600
C10—C21	1.479 (4)	C22—H22A	0.9600
N11—C12	1.424 (4)	C22—H22B	0.9600
N11—H11	1.01 (3)	C22—H22C	0.9600
C12—C13	1.379 (4)	C23—H23A	0.9600
C12—C17	1.380 (4)	C23—H23B	0.9600
C13—C14	1.381 (5)	C23—H23C	0.9600
C13—C22	1.505 (5)		
			
C6—C1—C2	121.3 (3)	C14—C15—H15A	119.6
C6—C1—N7	120.1 (3)	C15—C16—C17	121.0 (4)
C2—C1—N7	118.6 (3)	C15—C16—H16A	119.5
C3—C2—C1	118.1 (3)	C17—C16—H16A	119.5
C3—C2—C18	121.8 (4)	C12—C17—C16	117.4 (3)
C1—C2—C18	120.1 (3)	C12—C17—C23	121.2 (3)
C4—C3—C2	120.2 (4)	C16—C17—C23	121.4 (3)
C4—C3—H3A	119.9	C2—C18—H18A	109.5
C2—C3—H3A	119.9	C2—C18—H18B	109.5
C5—C4—C3	121.1 (4)	H18A—C18—H18B	109.5
C5—C4—H4A	119.5	C2—C18—H18C	109.5
C3—C4—H4A	119.5	H18A—C18—H18C	109.5
C4—C5—C6	120.8 (4)	H18B—C18—H18C	109.5
C4—C5—H5A	119.6	C6—C19—H19A	109.5
C6—C5—H5A	119.6	C6—C19—H19B	109.5
C1—C6—C5	118.5 (4)	H19A—C19—H19B	109.5
C1—C6—C19	120.5 (3)	C6—C19—H19C	109.5
C5—C6—C19	121.0 (4)	H19A—C19—H19C	109.5
C8—N7—C1	124.6 (4)	H19B—C19—H19C	109.5
C8—N7—H7	117 (2)	C8—C20—H20A	109.5
C1—N7—H7	119 (2)	C8—C20—H20B	109.5
N7—C8—C9	121.0 (3)	H20A—C20—H20B	109.5
N7—C8—C20	113.5 (3)	C8—C20—H20C	109.5
C9—C8—C20	125.5 (3)	H20A—C20—H20C	109.5
C8—C9—C10	127.7 (3)	H20B—C20—H20C	109.5
C8—C9—H9A	116.1	C10—C21—H21A	109.5
C10—C9—H9A	116.1	C10—C21—H21B	109.5
N11—C10—C9	120.0 (3)	H21A—C21—H21B	109.5
N11—C10—C21	113.9 (3)	C10—C21—H21C	109.5
C9—C10—C21	126.1 (3)	H21A—C21—H21C	109.5
C10—N11—C12	125.6 (4)	H21B—C21—H21C	109.5
C10—N11—H11	120 (2)	C13—C22—H22A	109.5
C12—N11—H11	115 (2)	C13—C22—H22B	109.5
C13—C12—C17	122.4 (3)	H22A—C22—H22B	109.5
C13—C12—N11	118.6 (3)	C13—C22—H22C	109.5
C17—C12—N11	118.9 (3)	H22A—C22—H22C	109.5
C12—C13—C14	117.5 (3)	H22B—C22—H22C	109.5
C12—C13—C22	120.4 (3)	C17—C23—H23A	109.5
C14—C13—C22	122.1 (4)	C17—C23—H23B	109.5
C15—C14—C13	120.8 (4)	H23A—C23—H23B	109.5
C15—C14—H14A	119.6	C17—C23—H23C	109.5
C13—C14—H14A	119.6	H23A—C23—H23C	109.5
C16—C15—C14	120.8 (4)	H23B—C23—H23C	109.5
C16—C15—H15A	119.6		
			
C6—C1—C2—C3	−0.9 (11)	C8—C9—C10—N11	−179.2 (3)
N7—C1—C2—C3	178.4 (6)	C8—C9—C10—C21	0.3 (5)
C6—C1—C2—C18	−179.1 (6)	C9—C10—N11—C12	0.3 (5)
N7—C1—C2—C18	0.2 (11)	C21—C10—N11—C12	−179.3 (3)
C1—C2—C3—C4	0.7 (12)	C10—N11—C12—C13	−92.5 (6)
C18—C2—C3—C4	178.9 (8)	C10—N11—C12—C17	90.3 (6)
C2—C3—C4—C5	−0.9 (14)	C17—C12—C13—C14	−2.5 (11)
C3—C4—C5—C6	1.2 (15)	N11—C12—C13—C14	−179.6 (6)
C2—C1—C6—C5	1.2 (10)	C17—C12—C13—C22	178.9 (6)
N7—C1—C6—C5	−178.1 (6)	N11—C12—C13—C22	1.8 (10)
C2—C1—C6—C19	−179.9 (6)	C12—C13—C14—C15	2.2 (12)
N7—C1—C6—C19	0.8 (10)	C22—C13—C14—C15	−179.3 (8)
C4—C5—C6—C1	−1.4 (12)	C13—C14—C15—C16	−1.6 (14)
C4—C5—C6—C19	179.8 (8)	C14—C15—C16—C17	1.2 (14)
C6—C1—N7—C8	−88.2 (6)	C13—C12—C17—C16	2.1 (10)
C2—C1—N7—C8	92.5 (6)	N11—C12—C17—C16	179.2 (6)
C1—N7—C8—C9	−0.8 (5)	C13—C12—C17—C23	−178.5 (6)
C1—N7—C8—C20	178.1 (3)	N11—C12—C17—C23	−1.4 (9)
N7—C8—C9—C10	179.4 (3)	C15—C16—C17—C12	−1.4 (11)
C20—C8—C9—C10	0.6 (5)	C15—C16—C17—C23	179.2 (7)

### Hydrogen-bond geometry (Å, °)

**Table:**

*D*—H···*A*	*D*—H	H···*A*	*D*···*A*	*D*—H···*A*
N11—H11···Cl1	1.01 (3)	2.12 (3)	3.115 (3)	170 (3)
N7—H7···Cl1^i^	1.00 (3)	2.12 (3)	3.110 (4)	170 (3)

Symmetry codes: (i) *x*, *y*, *z*−1.

## References

[bb1] Bourget-Merle, L., Lappert, M. F. & Severn, J. R. (2002). *Chem. Rev.* **102**, 3031–3066. 10.1021/cr010424r 12222981

[bb2] Brownstein, S., Gabe, E. J. & Prasad, L. (1983). *Can. J. Chem* **61**, 1410–1413.

[bb3] Carey, D. T., Cope-Eatough, E. K., Vilaplana-Mafé, E., Mair, F. S., Pritchard, R. G., Warren, J. E. & Woods, R. J. (2003). *Dalton Trans.* pp. 1083–1093.

[bb4] Dorman, L. C. (1966). *Tetrahedron Lett.* **7**, 459–464.

[bb5] Holland, P. L. & Tolman, W. B. (2000). *J. Am. Chem. Soc.* **122**, 6331–6332.

[bb6] Kuhn, N., Fahl, J., Fuchs, S. & Henkel, G. (2000). *Z. Naturforsch. Teil B*, **55**, 345–346.

[bb7] Lesikar, L. A. & Richards, A. F. (2006). *J. Organomet. Chem* **691**, 4250–4256.

[bb8] Macrae, C. F., Edgington, P. R., McCabe, P., Pidcock, E., Shields, G. P., Taylor, R., Towler, M. & van de Streek, J. (2006). *J. Appl. Cryst.* **39**, 453–457.

[bb9] Nagendran, S. & Roesky, W. H. (2008). *Organometallics*, **27**, 457–492.

[bb10] Park, K.-H. (2007). US Patent Appl. US 2007 191 638.

[bb11] Sheldrick, G. M. (2008). *Acta Cryst.* A**64**, 112–122. 10.1107/S0108767307043930 18156677

[bb12] Siemens (1996). *XSCANS* Siemens Analytical X-ray Instruments Inc., Madison, Wisconsin, USA.

[bb13] Stender, M., Wright, R. J., Eichler, B. E., Prust, J., Olmstead, M. M., Roesky, H. W. & Power, P. P. (2001). *Dalton Trans.* pp. 3465–3469.

